# Parent-reported health-related quality of life in pediatric childhood cancer survivors and factors associated with poor health-related quality of life in aftercare

**DOI:** 10.1007/s11136-023-03436-8

**Published:** 2023-05-19

**Authors:** Jana Winzig, Laura Inhestern, Verena Paul, Mona L. Nasse, Konstantin A. Krauth, Daniela Kandels, Stefan Rutkowski, Gabriele Escherich, Corinna Bergelt

**Affiliations:** 1 Department of Medical Psychology, University Medical Center Hamburg-Eppendorf, Martinistraße 52, 20246 Hamburg, Germany; 2 Department of Pediatrics, Pediatric Hematology and Oncology, Klinik Bad Oexen, Oexen 27, 32549 Bad Oeynhausen, Germany; 3 Swabian Children’s Cancer Center, Medical Faculty, University of Augsburg, Stenglinstraße 2, 86156 Augsburg, Germany; 4 Department of Pediatric Hematology and Oncology, University Medical Center Hamburg-Eppendorf, Martinistraße 52, 20246 Hamburg, Germany; 5 Department of Medical Psychology, University Medicine Greifswald, Walther-Rathenau-Str. 48, 17475 Greifswald, Germany

**Keywords:** Psycho-oncology, Pediatric cancer, Aftercare, Well-being, Parental assessment, Risk factors

## Abstract

**Purpose:**

Despite advances in cancer treatment, there is a prevalence of pediatric childhood cancer survivors still at risk of developing adverse disease and treatment outcomes, even after the end of treatment. The present study aimed to (1) explore how mothers and fathers assess the health-related quality of life (HRQoL) of their surviving child and (2) evaluate risk factors for poor parent-reported HRQoL in childhood cancer survivors about 2.5 years after diagnosis.

**Methods:**

We assessed parent-reported HRQoL of 305 child and adolescent survivors < 18 years diagnosed with leukemia or tumors of central nervous system (CNS) with the KINDL-R questionnaire in a prospective observational study with a longitudinal mixed-methods design.

**Results:**

In agreement with our hypotheses, our results show that fathers rate their children’s HRQoL total score as well as the condition-specific domains *family* (*p* = .013, *d* = 0.3), *friends* (*p* = .027, *d* = 0.27), and *disease* (*p* = .035, *d* = 0.26) higher than mothers about 2.5 years after diagnosis. Taking variance of inter-individual differences due to family affiliation into account, the mixed model regression revealed significant associations between the diagnosis of CNS tumors (*p* = .018, 95% CI [− 7.78, − 0.75]), an older age at diagnosis, (*p* = .011, 95% CI [− 0.96, − 0.12]), and non-participation in rehabilitation (*p* = .013, 95% CI [− 10.85, − 1.28]) with poor HRQoL in children more than 2 years after being diagnosed with cancer.

**Conclusion:**

Based on the results, it is necessary for health care professionals to consider the differences in parental perceptions regarding children’s aftercare after surviving childhood cancer. High risk patients for poor HRQoL should be detected early, and families should be offered support post-cancer diagnosis to protect survivors’ HRQoL during aftercare. Further research should focus on characteristics of pediatric childhood cancer survivors and families with low participation in rehabilitation programs.

## Introduction

Childhood cancer is a serious disease that affects more than 2000 children and adolescents each year in Germany [1]. Among children, the most frequent cancer diagnoses are leukemias and CNS tumors [1]. Thanks to advances in cancer treatment and improved diagnostic methods, the 15-year survival rate is 82% across all pediatric cancer diagnoses in developed countries [1, 2]. Despite the improved survival rates, cancer and the corresponding multifaceted treatment can have far-reaching consequences for a child, causing physical and mental burden, even after the end of treatment [3]. In Germany, every pediatric childhood cancer survivor continues to attend regular oncological aftercare until the age of 18, even after the acute aftercare has already been completed. Especially with more time after the end of treatment, many clinics provide late effect-oriented aftercare. Implementation is facilitated by children's hospitals, which integrate many specialties in addition to pediatric oncologists [4].

To measure children’s health outcomes, general well-being and everyday functions, the multidimensional construct health related quality of life (HRQoL) was utilized [5]. According to the international guidelines, it is common to assess HRQoL in childhood cancer survivors’ aftercare [6]. Nevertheless, HRQoL is less focused in aftercare than in acute care and empirical evidence is not entirely consistent. While a review by Shin and colleagues reported similar or even higher HRQoL scores for most survivors compared to healthy controls [7], various other studies illustrated the remarkably complex and powerful impact of childhood cancer on psychosocial and physical HRQoL [8–11], even in long-term childhood cancer suvivors [12, 13]. Additionally, a systematic review showed that childhood cancer survivors experience lower levels of mental well-being, reduced positive mood, and lower self-esteem as well as increased anxiety and physical and sleeping difficulties compared to healthy controls and population norms [9].

In Germany, as in most Western countries, parents are surrogates for their children as long as they are not old enough to make autonomous decisions [14]. In aftercare of childhood cancer, the parental role is important, and further implications for children’s health care often result from parental involvement [15]. Due to the different roles of mothers and fathers in coping with the disease, they might perceive their children’s HRQoL during aftercare differently. Heterogenous perceptions may result in different implications for aftercare. Therefore, it is necessary to identify how mothers and fathers assess their children’s HRQoL.

Early identification of vulnerable children with a high risk for decreased HRQoL during aftercare is essential to allow health care professionals (HCPs) to offer preventative care and help where it is needed. In Germany, pediatric cancer survivors and their family members can participate in a 4-week family-oriented rehabilitation (FOR) stay to recover from disease and to regain psychological and physical well-being [16]. Several risk factors for poor HRQoL in children and adolescent long-term childhood cancer survivors after completion of cancer treatment have already been identified: Type of cancer and a higher number of applied treatment modalities, chronic symptoms, late effects, and demographic differences including higher age, female gender, lower education, and lower household income [7, 13]. Survivors of central nervous system (CNS) tumors reported poorer physical and psychological functioning compared to leukemia survivors [17]. However, the studies primarily focused on either long-term survivors or patients immediately after and during treatment, while studies focusing on the aftercare period (up to 5 years after end of treatment) are scarce.

Hence, this study aims to investigate how mothers and fathers assess their child’s HRQoL, assuming that fathers rate their child’s HRQoL higher than mothers [18]. Additionally, the objective of the study was to evaluate risk factors for poor HRQoL in children 2.5 years after being diagnosed with cancer and during aftercare when survivors are followed-up regularly. To provide information on vulnerable patients at an early stage after completion of cancer treatment and without high additional effort, we focused on sociodemographic and disease-specific factors. Moreover, we focused on the most frequent cancer diagnoses in children, leukemias and CNS tumors [1]. Thus, the present study contributes to identifying high-risk patients at an early stage. At the same time, it clarifies potential differences in parental assessments regarding child HRQoL after surviving cancer.

## Methods

### Design

The study is a secondary analysis of the quantitative data from a prospective observational study with a longitudinal mixed-methods design [19]. The study was approved by the Ethics Committee of the Medical Chamber of Hamburg (Number PV5277).

### Participants and procedure

Study participants, identified through pediatric cancer study registries and a rehabilitation clinic, were surveyed through standardized questionnaires either after the end of intensive treatment or at the beginning of the rehabilitation (baseline) and at the 12–18 month follow-up. We included parents (biological parents and other caregivers) whose child had been diagnosed with a CNS tumor or acute leukemia before the age of 18 and whose acute cancer treatment was completed. We aimed to assess both parents individually. We excluded participants with high mental burden (if participation in the study would have been an additional burden), cognitive limitations, insufficient German language skills and those who refused to participate in the survey. Longitudinal data was collected between July 2016 and December 2020 using parent-reported questionnaires in two settings: (1) The patients’ clinic was informed about the study via study registries (International HIT-MED Registry, ClinicalTrials.gov Identifier: NCT02417324; COALL 08–09 study, ClinicalTrials.gov Identifier: NCT01228331; SIOP-LGG 2004 study, ClinicalTrials.gov Identifier: NCT00276640) at the end of intensive cancer treatment. Parents were informed about the study by health care providers in the clinics and were given a consent form to contact. Families who agreed to be contacted received written information about the study, a consent form for participation, and questionnaires from the research institute. (2) Families in a cooperating rehabilitation clinic were informed about the study at the beginning of the rehabilitation program and received a consent form and the questionnaires, if interested in study participation. Further information on the recruitment process are provided in the study protocol [19].

### Outcome

According to the study aims, the primary outcome was children’s health-related quality of life (HRQoL) 12–18 months after treatment, measured with the KINDL-R questionnaire for caregivers at follow-up [5]. The KINDL-R was designed to assess children’s HRQoL and consists of seven subscales: physical well-being, mental well-being, self-esteem, family, friends, functioning in everyday life (school or preschool/kindergarten) and disease. The seven subscales are transformed to a range 0 to 100. Additionally, a total score based on all items can be calculated. Parents rated their child’s quality of life in the past seven days on 5-point Likert scales extending from *never* (1) to *all the time* (5). Higher values indicate higher HRQoL. The KINDL-R has proven to be reliable and valid [20, 21].

To assess participants’ socioeconomic status (SES), we used the Winkler Index which comprises three status variables (education, occupation, and income) [22]. Further participants’ variables (e.g. marital status) at baseline and childrens’ medical information (e.g. diagnosis, treatments received) at follow-up were collected by tools developed by the authors themselves [19].

### Statistical analyses

Descriptive statistics such as frequencies, means and standard deviations, medians and ranges were calculated. Unpaired *t*-test was performed to compare means of parental assessment of child’s HRQoL between mothers and fathers. We assumed that HRQoL (measured with the KINDL-R) 12–18 months after the end of cancer treatment can be predicted by a number of baseline variables. Due to clustered data structure regarding family affiliation, resulting from the assessment of two members from the same family, a linear mixed regression was modeled, including family affiliation as random effect. We confirmed that our outcome was normally distributed. To analyze predictors for the continuous outcome (HRQoL), measured by parent-reported KINDL-R, we first conducted univariate analyses and calculated a correlation table including all variables of interest (KINDL-R total score, children’s age at follow-up, children’s and parent’s gender, family’s SES, diagnosis (CNS tumor vs. Leukemia), patient age at diagnosis, time since diagnosis, number of applied treatment modalities and participation in rehabilitation). Depending on the recruitment path, the diagnosis and time since diagnosis were either parent-reported or reported by the physicians in the rehabilitation clinic. Remaining variables were parent-reported. Independent variables were included in the multivariate analysis if they were significantly correlated with the outcome at an alpha level of 0.05. Although “children’s and parent’s gender” and “family’s SES” were not significantly correlated with the outcome, we included these variables because previous studies have found significant associations between these variables and HRQoL [7, 13]. To avoid multicollinearity, we excluded variables that were highly correlated (r ≥ 0.7) with other variables and decided to include the diagnosis-related variable. Therefore, we excluded “number of applied treatment modalities” and included “diagnosis (CNS tumor vs. Leukemia)”. We also excluded “children’s age at follow-up” and included “patient age at diagnosis”. Finally, independent variables were included as fixed factors in our linear mixed model. The approach allows to include random effects in addition to fixed effects [23], so that we were able to control for family affiliation. Dummy coded variables were utilized when necessary and an alpha level of 0.05 was applied. All analyses were performed using IBM SPSS Statistics 27.

## Results

There were 899 families potentially eligible to participate in the study. If possible, we included both parents within a family. Five hundred and twenty-seven families that had been identified as eligible via the study registries could not participate either because they met the exclusion criteria or because the HCPs in the clinics could not inform them about the study. Reasons for non-participation for the remaining 60 families that had been identified as potentially eligible via the rehabilitation clinic were: No interest (*n* = 21), insufficient German language skills (*n* = 14), physical/and or mental burden (*n* = 12), cognitive limitations (*n* = 3), or not specified (*n* = 10). A total of 312 families participated in the survey. Due to missing signed consent forms (*n* = 2), missing parental questionnaires (*n* = 2), a wrong diagnosis (*n* = 2), or incorrectly answered questionnaires because of limited German language skills (*n* = 1), 7 families were subsequently excluded from the analyses. Therefore, due to robust estimators of mixed models, a total of 516 valid parental questionnaires of 305 families at baseline could be included in the mixed models (initial participation rate: 57.4%). In 211 families, both mother and father completed the baseline questionnaires. Two hundred and ninety-four parents of 172 childhood cancer survivors also completed the 12–18 month questionnaires at follow-up and could be included in the t-test analysis.

### Sample description

Characteristics of the parental study population are shown in Table 1. The majority of respondents are birth mothers (55.4%) or other female caregivers (2.5%), married (77.2%) and German native speakers (85.4%). Most of participating families have a medium (46.1%) or high (38.3%) SES according to Winkler and Stolzenberg (1998) and two to three children (71.2%) (Table 1).

**Table 1:** Sociodemographic characteristics of *n* = 516 parents of *n* = 305 childhood cancer survivors after end of intensive treatment (baseline)

Characteristics	*n* = 516	%
*Role for affected child		
Mother	299	57.9
Father	217	42.1
Native language^a^		
German	437	85.4
Other	75	14.6
Marital status^b^		
Never married	74	14.4
Married	396	77.2
No longer married	42	8.2
Widowed	1	0.2
**SES^c^		
Low	78	15.6
Medium	231	46.1
High	192	38.3
Number of children^b^		
1	108	21.1
2–3	365	71.2
> 3	40	7.8

^a^4 missings

^b^3 missings

^c^15 missings

*Summarising birth parents and other caregivers; **Stratification index according to Winkler & Stolzenberg (1998)

General and clinical characteristics of *n* = 305 childhood cancer survivors are displayed in Table 2. The male children accounted for 55.1% of the sample; mean age at baseline was 7.3 years (*SD* = 4.3). Leukemia was more frequent (62%) than CNS tumors (38%). The average survivor was 5.5 years old at diagnosis (range, 0–17 years with 71.6% diagnosed before the age of 8) and 2.5 years after diagnosis at the time of study inclusion (range, 1–13 years). The majority experienced one (45.7%) or between three to five (31.3%) treatments (Table 2).

**Table 2:** Sociodemographic and medical characteristics of *n* = 305 childhood cancer survivors

	*n* = 305	%
Sex^a^		
Boys	167	55.1
Girls	136	44.9
Diagnosis		
Leukemia	189	62
CNS tumor	116	38
Age at baseline, years^a^		
0–1	8	2.6
2–8	186	61.4
> 9	109	36
Age at diagnosis, years^a^		
0–1	52	17.2
2–8	181	59.7
> 9	70	23.1
Treatment modalities^b^		
Surgery	156	51.3
Chemotherapy	276	90.8
Radiation	91	29.9
Other treatment (e.g., stem cell transplantation, alternative medicine)	33	10.9
Number of treatments^b^		
1	139	45.7
2	56	18.4
3–5	95	31.3
> 6	14	4.6
Participation in rehabilitation^c^		
Yes	233	91.4
No	22	8.6
*Age at follow-up, years		
2–10	118	67.8
11–19	56	32.2
*Time since diagnosis at follow-up, years^b^		
1	19	11
2–3	129	74.6
> 4	25	14.4

^a^2 missings

^b^1 missing

^c^50 missings

*Follow-up-characteristics for *n* = 174 childhood cancer survivors

### Children’s HRQoL rated by their parents

Child’s HRQoL in the social domain *friends* was rated lowest (68.43 ± 18.75) while the *disease*-related domain was rated highest (82.08 ± 16.74) by parents. The comparison of parental assessment of child’s HRQoL (KINDL-R scores) between mothers and fathers is presented in Table 3. The mean HRQoL score assessed by parents was 74.07 ± 13.18, and results revealed that fathers rate the HRQoL of their children significantly higher than mothers on the domains *family* (*p* = 0.013), *friends* (*p* = 0.027), *disease* (*p* = 0.035), and in the *total* (*p* = 0.049) HRQoL score.

**Table 3:** Descriptive statistics for parent-reported HRQoL for *n* = 294 mothers and fathers of *n* = 172 childhood cancer survivors 2.5 years after diagnosis

	Total *n* = 294	Mothers *n* = 169	Fathers *n* = 125	*t*-value, *p*-value	Cohen’s *d*
HRQoL domains (KINDL-R)					
Physical^a^	72.26 ± 22.45	70.96 ± 22.91	74.06 ± 21.75	n.s	
Mental^b^	78.32 ± 15.43	77.63 ± 16.03	79.29 ± 14.57	n.s	
Esteem^a^	71.49 ± 15.33	70.56 ± 15.88	72.81 ± 14.49	n.s	
Family^c^	78.81 ± 13.86	77.10 ± 14.43	81.18 ± 12.70	t = − 2.492, *p* = .013	*d* = 0.3
Friends^a^	68.43 ± 18.75	66.37 ± 19.84	71.34 ± 16.73	t = − 2.229, *p* = .027	*d* = 0.27
School^d^	75.38 ± 16.89	74.70 ± 17.10	76.34 ± 16.61	n.s	
Disease^e^	82.08 ± 16.74	80.30 ± 17.06	84.54 ± 16.04	t = − 2.119, *p* = .035	*d* = 0.26
Total^c^	74.07 ± 13.18	72.77 ± 13.74	75.88 ± 12.89	t = − 1.981, *p* = .049	*d* = 0.23

The average overall parental-reported value in the representative healthy control sample (*n* = 14,836) aged 3–17 years is *M* = 76.9, 95% CI [76.7, 77.1] [24]

Missing value, if less than 70% of the items of the subscale or total score were answered, parental rates for HRQoL missing for:

^a^5 participants (1.7%)

^b^3 participants (1%)

^c^4 participants (1.4%)

^d^30 participants (10.2%)

^e^9 participants (3.1%)

### Predictors of HRQoL in children two years after end of acute treatment

We used a mixed model to estimate the parent-reported HRQoL in children after surviving childhood cancer 18–24 months after diagnosis. After accounting for inter-individual differences due to family affiliation, the mixed model regression revealed significant associations between the diagnosis of leukemia and higher HRQoL in contrast to the diagnosis of a CNS tumor, lower age at diagnosis and participation in a rehabilitation program. An intraclass correlation coefficient (ICC) of 0.54 suggested moderate reliability in the influence of family affiliation on children’s HRQoL reported by their parents or other caregivers (Table 4) [25].

**Table 4:** Mixed Model Estimates for predicting parent-reported HRQoL in childhood cancer survivors 18–24 months after diagnosis

	Est	*SE*	*t*	*p*	95% CI	
					lower	upper
Intercept	86.15	4.23	20.353	** < .001**	77.81	94.50
Fixed effects						
Female child	0.77	1.78	0.431	.667	− 2.74	4.27
Female parent/ caregiver	− 1.45	1.07	− 1.347	.180	− 3.57	0.68
Socioeconomic status ^a^	0.06	1.19	0.319	.750	− 0.31	0.43
Diagnosis (Leukemia) ^b^	− 4.26	1.78	− 2.394	**.018**	− 7.78	− 0.75
Age at diagnosis	− 0.54	0.21	− 2.556	**.011**	− 0.96	− 0.12
Time since diagnosis	− 1.03	0.54	− 1.898	.059	− 2.10	0.04
Participation in rehabilitation ^c^	− 6.06	2.43	− 2.499	**.013**	− 10.85	− 1.28
Random effects						
Residual σ^2^	.46					
ICC	.54					

^a^SES index according to Winkler

^b^Leukemia vs. CNS tumor

^c^yes/no

Residual *σ*^*2*^: Residual variance, *ICC*: Intraclass correlation coefficient, *Est*: Estimations, *SE*: Standard error of fixed effects, *CI*: Confidence interval

## Discussion

The purpose of this study was to investigate parent-reported health-related quality of life (HRQoL) in children and adolescents after surviving childhood cancer.

Firstly, we wanted to identify differences between mothers and fathers in their assessment of their children’s HRQoL. Previously, only a few studies investigated parent-reported child HRQoL [26–28]. However, parent-reported HRQoL of early childhood cancer survivors has not been examined before. Many health-related decisions are made on parental reports because they can provide HCPs with useful information about their children. Thus, especially for younger children, parents’ assessment play a crucial role in health care and health-related decisions [29]. Evidence shows that reports of a child’s HRQoL from parents and children are highly consistent [30, 31]. Therefore, parental reports as applied in the current study can be obtained with reasonable confidence in case a child is too young or too sick to provide self-report. Our results show that fathers rate their children’s HRQoL total score and the condition-specific domains *family*, *friends*, and *disease* higher than mothers. Results are partly in line with research on other chronic conditions such as epilepsy and esophageal atresia: While Kalyva and Melonashi did not find any differences between maternal and paternal reports of child HRQoL, Witt and collegues reported that fathers rated child HRQoL scores in the condition-specific *social* domain higher than mothers [26, 28]. It is important to consider that mothers of children with cancer might experience higher levels of distress than fathers [32, 33]. Different roles of mothers and fathers in coping with the disease might affect their parental experience and their perception of childs’ HRQoL. Mothers seem to be more involved in supporting their child by accompanying the child during hospital stays, whereas fathers are more likely to stay at home or return to work after a little while [34]. At the same time, mothers’ own well-being and worries may impact their ratings of their child’s HRQoL being lower [33, 35]. Consequently, it can be expected that factors such as parental functioning also have an impact on our findings. Current literature identified modifiable factors associated with parental ratings of their child’s HRQoL. Fear of cancer relapse and fear of late effects, as well as parental resilience, may play a role in reporting HRQoL in pediatric survivors [36]. Parents take an important role in aftercare of childhood cancer and implications for children’s health care often result from parental report [15]. Therefore, it is necessary keeping in mind the differences in parental perceptions when it comes to children’s aftercare after surviving childhood cancer because heterogeneous perceptions could result in different implications for aftercare.

Secondly, we wanted to identify baseline factors associated with a risk for poor HRQoL during aftercare, about 2.5 years after diagnosis. Compared to reference data from the German Child and Adolescent Health Survey (KiGGS), childhood cancer survivors in this study sample were slightly below the average of healthy peers (healthy controls: *M* = 76.9; childhood cancer survivors: *M* = 74.1) [24]. In our linear model, family affiliation as random effect explained a moderate level of information on group level (ICC = 0.54). Moreover, our analyses identified the diagnosis of CNS tumor, an older age at diagnosis, and non-participation in a rehabilitation program as risk factors associated with poor HRQoL in children approximately 2 years after the end of intensive cancer treatment. These factors may help HCPs to identify patients at risk early in the disease trajectory. Our findings are in line with previous findings reporting a lower overall HRQoL in survivors of CNS tumors compared to leukemia and other childhood cancer survivors [37, 38]. Authors of the Childhood Cancer Survivor Study also identified adult brain tumor survivors as vulnerable patients for poor HRQoL. Especially, high intense treatments such as cranial radiation and/or surgery are associated with lower HRQoL after end of treatment [10, 36, 39]. However, most of these studies investigated HRQoL of (young) adult childhood cancer (AYA) survivors instead of pediatric survivors [40–44]. Still, it becomes clear that especially the HRQoL of CNS tumor survivors needs to be more protected. We advocate more early interventions that support survivors individually and on multiple levels, e.g., cognitively, socially, emotionally. Our results indicate that lower HRQoL already shows during the first 2 years after the end of treatment. Additionally, our analyses showed that age was associated with HRQoL. An older age at diagnosis predicted poorer HRQoL at follow-up. It is possible that childhood cancer survivors who were diagnosed in their first years of life hardly remember anything and thus have less burden. Additionally, the treatment of childhood cancers tends to be shorter and less intensive than that of adolescents, and there are developmental and psychosocial aspects of adolescence that the treatment might affect [39, 45]. Our findings display the need for age-related support offers and are in line with previous research that focused on HRQoL and the positive and negative impact of cancer in AYA survivors [39, 46]. Moreover, previous research has found that female sex, lower socioeconomic status, and more time since diagnosis are associated with poor HRQoL in aftercare, whereas we found no association between these predictors and outcome [44, 47–49]. Our findings indicate that most partcipants have participated in a rehabilitation program. Participation in a FOR was associated with better HRQoL at follow-up in our sample, indicating that FOR is beneficial for survivors and their families. FOR supports survivors and their families in reintegrating into daily life, gaining physical strength, and having peer contact [50]. We can assume that survivors and their families, particularly those at high risk for negative long-term consequences, may also benefit from FOR. However, several barriers can hinder the families’ participation in FOR, including concerns about work or school disruption, the effort involved in submitting an application, cultural and language barriers, as well as psychological barriers [51–53].

Therefore, it is important to make information about FOR easily accessible and to inform parents and families about the potential benefits of a rehabilitation program for improving HRQoL after intensive treatment ends.

## Limitations

When interpreting the results of our analyses the following limitations should be considered. Firstly, due to data protection regulations, a personal contact of the parents was not possible, and parents were recruited via study registries and a rehabilitation clinic. Within this study design, we could not conduct a non-responder analysis which might limit the representativeness of the sample. Secondly, the current study is a secondary analysis of a main study which focused on the role of rehabilitation measures supporting families in the reintegration into daily life, school, and work. It should be kept in mind that all families were offered to participate in a FOR. Therefore, we cannot control for any selection bias that might affect our results. Thirdly, our outcome HRQoL in childhood cancer survivors was calculated only by parental proxy report. For our second aim, it would be even more valid to obtain data from both informants, the children themselves and their parents. Therefore, it is possible that we under- or overestimated the children’s true HRQoL. However, it can also be considered as a strength of our study that we included children of all age groups and thus also young children who are too young to self-report. Although obtaining reports from two parents is preferable, this was not always possible. Nevertheless, in most cases, both the maternal and the paternal report was available so that we included family affiliation as random effect. Additionally, the study design was predominantly questionnaire-based and appropriate German language skills were necessary to enter the study. Additionally, there were less CNS tumor survivors in our sample who tend to have an increased risk for higher burden compared to leukemia survivors because of cranial radiation [54]. At the same time, the review by Cantrell and collegues shows that female childhood cancer survivors have a higher risk for poor HRQoL compared to male childhood cancer survivors [54]. Therefore, we cannot account for these selection biases and over- or underestimation of burden is possible [55]. Lastly, due to the small number of families who did not participate in a FOR, results regarding the impact of the rehabilitation program can only be interpreted to a limited extent.

Despite the several limitations, the findings of the present study are important in the context of providing health care for pediatric childhood cancer survivors in an early phase of survivorship. Due to the comparatively large sample size, the longitudinal design, and recruitment from both a rehabilitation clinic and study registries, our results can be considered as reliable. They showed differences in parental reports of their childs’ HRQoL, with fathers rating their child’s HRQoL higher than mothers. Additionally, our sample provides evidence based on a large sample of childhood cancer survivors that several risk factors such as the diagnosis of CNS tumor, an older age at diagnosis, and non-participation in a rehabilitation program are associated with a poorer HRQoL in children after surviving childhood cancer.

### Implications for future research

Results of the study suggest that support offers and recommendations for aftercare should be tailored to specific target- and risk groups, especially to those who carry multiple risk factors. Moreover, a more heterogenous sample with participants from different countries would provide even more generalizable results. Our findings indicate benefits of participation in rehabilitation measures to lower the risk for poor child HRQoL during follow-up. Further research should investigate characteristics of pediatric childhood cancer survivors and their families who tend not to participate in rehabilitation measures or other aftercare programs and the factors influencing that. For example, relevant research questions may comprise reasons for non-participation in rehabilitation and differences between participants and non-participants.

## Conclusion

It is necessary to keep in mind identified differences in parental perception, because parents take an important role in aftercare of childhood cancer and their perceptions can implicate children’s health care decisions. Early identification of patients with several risk factors is necessary to initiate adequate support offers during aftercare. HCPs and families should be educated and made aware of risk factors. Moreover, support should be offered to enable informed decisions in favour of pediatrics HRQoL after surviving childhood cancer.

## Data Availability

Additional anonymized data found in manuscript are available from the Corresponding Author upon reasonable request.
